# Anticancer Effect of Mycotoxins From *Penicillium aurantiogriseum*: Exploration of Natural Product Potential

**DOI:** 10.1155/ijm/5553860

**Published:** 2024-12-05

**Authors:** Assia Bouhoudan, Joaira Bakkach, Mustapha Khaddor, Nadira Mourabit

**Affiliations:** ^1^Department of Biology, Laboratory of Research and Development in Engineering Sciences, Faculty of Sciences and Techniques of Al-Hoceima, Abdelmalek Essaadi University, Tetouan 93000, Al-Hoceima, Morocco; ^2^Department of Biology, Higher Institute of Nursing Professions and Health Techniques of Tetouan, Al-Hoceima 93000, Morocco; ^3^Regional Center for Careers Education and Training of Tangier, Tangier 90000, Morocco

**Keywords:** anticancer, mycotoxins, natural substances, *P. aurantiogriseum*, therapeutic agent

## Abstract

Research into biologically natural substances with antitumor properties, known for their potential to induce fewer side effects and exhibit specificity toward cancerous cells, remains imperative. The pressing demand for novel agents in cancer therapy underscores the intensive investigation of natural products from microorganisms. *Penicillium aurantiogriseum*, frequently isolated from food and feed, emerges as a promising candidate against pathogenic bacteria and fungi. This species harbors numerous mycotoxins that warrant extensive clinical study due to their potential in cancer treatment. Identifying mycotoxins with anticancer properties produced by *P. aurantiogriseum* could unveil novel therapeutic targets and enrich the pharmacological landscape. This review provides a comprehensive overview of the utilization of *P. aurantiogriseum* mycotoxins in cancer research and elucidates therapeutic agents' advantages and limitations. *P. aurantiogriseum* produces at least 15 mycotoxins with potent anticancer effects mediated through diverse mechanisms, including enzyme inhibition (e.g., pseurotin), induction of apoptosis (e.g., auranthine, aurantiamides A, aurantiomides A-C, penicillic acid, penitrem, verrucisidinol, acetate verrucosidinol, and chaetoglobosin A), and cell-cycle arrest (e.g., anicequol, aurantiamine, and Taxol). Although certain mycotoxins, such as Taxol, Anacin, and Compactin, are used in commerce, many others remain relatively unexplored. The mycotoxins derived from *P. aurantiogriseum* hold considerable potential for cancer treatment, offering novel therapeutic avenues and enhancing current treatments through synergistic combinations and advanced delivery systems.

## 1. Introduction

Cancer is one of the deadliest diseases worldwide [1]. According to the 2024 World Cancer Report published by the World Health Organization (WHO), there were approximately 20 million new cancer cases and 10 million deaths in 2022 [2]. The global cancer burden is projected to rise to 28.4 million cases by 2040, driven by demographic changes and increasing risk factors [3]. Developing novel therapeutic strategies is crucial to combating this complex disease. Pharmacotherapy, primarily chemotherapy, is one of the main treatment approaches currently used. Over 50 years, around 5 million natural and synthetic chemical compounds have been evaluated for anticancer efficacy [4, 5]. Data from the U.S. Food and Drug Administration (FDA) and the WHO indicate that approximately 150–200 cancer treatment agents (both synthetic and natural) are approved for clinical use globally, including conventional chemotherapy drugs, immunotherapies, and other targeted agents [6–8]. The approved drugs comprise standard cytotoxic chemotherapy agents and innovative treatment alternatives. Conventional treatments have frequently encountered challenges such as drug resistance and side effects, which highlight the urgent need for alternative approaches.

The urgent need for innovative cancer treatment drives a thorough exploration of natural compounds originating from microorganisms. Within the numerous organisms exploited in the pharmaceutical industry, fungal species belonging to the *Penicillium* genus stand out due to their varied and potent secondary metabolite, both quantitatively and qualitatively [9].

*Penicillium aurantiogriseum* (*P. aurantiogriseum*) is a notable species within this genus, ubiquitous in terrestrial and marine environments [10]. It is recognized as a prolific source of biologically active substances, with mycotoxins exhibiting a range of harmful and beneficial effects on human and animal health [11]. Numerous studies have highlighted the importance and attractiveness of *P. aurantiogriseum* mycotoxins [12]. For instance, Zeng et al. demonstrated that auranomide B, isolated from *P. aurantiogriseum,* exhibited potent antiproliferative activity against human lung cancer cells [13]. Similarly, Kalinina et al. reported that auranthine from *P. aurantiogriseum* inhibits the proliferation of human lung cancer cells [14]. This growing interest in marine-derived fungi is linked to their bioactive secondary metabolites, which exhibit diverse biological activities, including promising anticancer properties [15].

Furthermore, Park et al. highlighted the selective cytotoxicity of verrucosidin from *P. aurantiogriseum* against colon cancer cells, demonstrating striking [16]. Tian et al. supported this by reporting that chaetoglobosin A induces apoptosis (programmed cell death) and exhibits immunosuppressive activity in cancer cells [17]. These findings collectively highlight the significant potential of *P. aurantiogriseum* as a source of novel anticancer drug development compared to other molds. The diverse pharmacological response of *P. aurantiogriseum* mycotoxins has attracted the attention of medicinal chemists, prompting further exploration of this species for its multifaced potential.

This review examines the potential of mycotoxins produced by *Penicillium aurantiogriseum* as novel anticancer agents by evaluating their biological effects on various cancer cell lines. Through this analysis, we aim to provide valuable insights for researchers, clinicians, and pharmaceutical developers interested in leveraging these compounds for therapeutic applications. In addition, our findings may inform future studies on mycotoxins from marine fungi and their role in cancer treatment strategies.

## 2. *Penicillium aurantiogriseum*

*P. aurantiogriseum* is a species of fungus belonging to the genus *Penicillium*, family Trichocomaceae, order Eurotiales, subclass Eurotiomycetes, class Pezizomycotina, phylum Ascomycota, and kingdom fungi [15]. [18]. First described by Dierckx in 1901 [19], it is one of 225 species within the *Penicillium* genus (the primary group of the Trichocomaceae family). It is known to be one of the most toxic species [20]. *P. aurantiogriseum* is frequently isolated from various foods [21].

The marine environment presents conditions such as high pressure, increased salinity, oxygen deficiency, and limited nutrition availability that are unique and ideal for fungus growth. Nevertheless, *P. aurantiogriseum* has demonstrated the ability to grow in this environment, including permafrost sediments from the Arctic and Antarctic [10, 22], which suggests its properties as a facultative marine fungus. *P. aurantiogriseum* exhibits resilience to various stress factors related to nutrients and growing conditions.

Recent studies indicate that over 1000 new natural products have been characterized by marine fungi, with many being closely related to those from terrestrial sources [23]. In recent years, marine fungi, including *P. aurantiogriseum,* have produced several metabolites demonstrating significant anticancer effects by inhibiting key enzymes, triggering apoptosis, or promoting growth arrest [24].

Amid the diverse array of natural products exhibiting anticancer potential, *P. aurantiogriseum* presents a remarkable species for its unparalleled capacity to produce diverse mycotoxins with potent anticancer properties. Though numerous fungi from terrestrial and marine environments are recognized for their potential to produce anticancer compounds, *P. aurantiogriseum* stands out as a prolific source, which makes it a valuable resource for developing novel and effective anticancer therapies.

## 3. Mycotoxins of *Penicillium aurantiogriseum*

Mycotoxins are hazardous natural substances excreted by various mold species, which can develop on a range of foods and animal feed under the right temperature and humidity conditions. This poses significant threats to both human and animal health. Several studies have investigated the *P. aurantiogriseum* mycotoxins [25–27]. These mycotoxins exhibit diverse biological activities and hold the potential for discovering novel therapeutic agents with antibiotic, antiviral, antifungal, anticancer, and cholesterol-lowering properties [9, 24]. In addition, some mycotoxins may target specific diseases [9].

Certain mycotoxins produced by *P. aurantiogriseum* have demonstrated antitumor effects in preclinical studies and are being explored for their potential as anticancer agents [28]. Some of these compounds are already available on the market [29–31], while others have received limited attention.  Table 1 highlights the potential of *P. aurantiogriseum* mycotoxins for developing new therapeutic agents. These range from beneficial properties such as antibiotic, antiviral, and anticancer effects (e.g., auranomide B and chaetoglobosin A) to cholesterol-lowering properties (e.g., Compactin) and antioxidant activity (e.g., orsellinic acid). In addition, Anacin offers pain relief and fever reduction, while Taxol, a well-established anticancer drug, is also produced by *P. aurantiogriseum*.

Mycotoxins with promising bioactivities such as cytotoxicity (auranthine, aurantiamides, and verrucosidinol) suggest potential for further research and development of novel therapeutic agents. While some mycotoxins show promising therapeutic potential, others may pose health risks. For example, carcinogenic mycotoxins such as penicillic acid highlight the need for further research to fully understand their mechanisms of action, potential side effects, and safety before considering any *P. aurantiogriseum-*derived mycotoxin for human applications.

### 3.1. Nature of Mycotoxins

Toxigenesis, the production of mycotoxins by molds, does not have a biochemical impact on fungi's growth and development and plays no discernible role in the economics of the living cells that synthesize these compounds [49]. However, it appears to be a response of fungi to adverse environmental factors, such as excessive or inadequate warmth and humidity [50].

### 3.2. Synthetic Pathways for *P. aurantiogriseum* Mycotoxins

**Figure 1:**
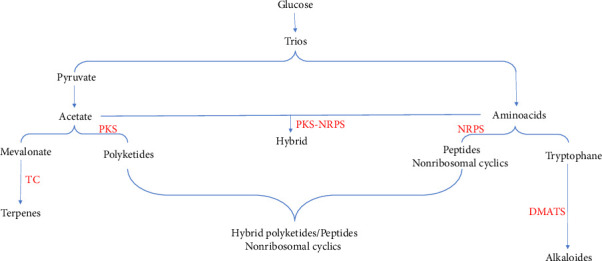
Representation of metabolic origins of different mycotoxins. Mycotoxins can be classified into four categories (nonribosomal cyclic peptides, polyketoacids, terpenes, and hybrids) metabolized from glucose. Abbreviations: DMAST, dimethylallytransferase; NRPS, nonribosomal peptide synthetase; PKS, polyketide synthase; TC, terpenes cyclase.

Mycotoxins, as low molecular weight, are characterized by their varied structural and chemical origins, which correspond to their specific biosynthetic pathways. The mycotoxins produced by *P. aurantiogriseum* do not constitute a separate class of chemicals; instead, they originate from three primary sources: amino acids, polyketoacids, and terpenes [51, 52]. This diversity in structure stems from various chemical reactions that occur during their biosynthesis, including cyclization, aromatization, glycosylation, hydroxylation, and epoxidation [53]. Consequently, mycotoxins can be classified into four major groups: terpenes, nonribosomal cyclic peptides, polyketoacids, and hybrids. Given its ability to produce various mycotoxins, *P. aurantiogriseum* demonstrates a variety of biosynthetic pathways in its metabolism [9, 54–58]. The multiple metabolic sources of *P. aurantiogriseum* mycotoxins are illustrated in Figure 1 and detailed in Table 2.

### 3.3. Gene Cluster and Regulation

**Figure 2:**
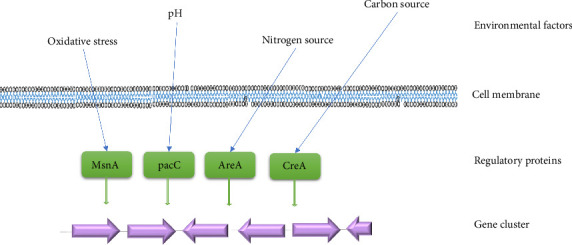
Possible connections between environmental signals and regulatory elements.

The biosynthesis pathway for mycotoxins, whether polyketide, terpene, or peptide, involves a series of transformation enzymes. To ensure an adequate rate of final product formation and to prevent degradation by potential parasitic reactions, the product of each enzymatic reaction in a metabolic chain must be rapidly transferred to the following enzyme. Consequently, the genes encoding these enzymes are activated simultaneously. This coordinated action is facilitated by the “clustering” of the genes coding for these transformational enzymes [58, 60] (Figure 2). Clusters containing all (or nearly all) of the genes involved in the biosynthetic pathway of the metabolites are responsible for regulating their production. Among these genes, one usually encodes a regulatory factor, typically functioning as an activator, which enhances the expression of all adjacent genes in response to a stimulus. Nevertheless, some biosynthetic clusters lack a regulatory factor, suggesting that its presence is not always required [61].

The existence of a gene cluster does not necessarily imply that a particular fungal species can produce the associated secondary metabolite. Some clusters may be detectable but exist in a “skeleton” state, meaning they lack all of the genes encoding the necessary enzymes for a complete biochemical pathway, thereby preventing production. Bioinformatics analyses indicate that it cannot definitively predict the number of families of secondary metabolites produced by a fungal species. Thus, it should be complemented by genetic and metabolomic analyses to provide a comprehensive understanding of the metabolite landscape [62].

In contrast to other significant mycotoxins such as aflatoxins and trichothecenes, the genetics of mycotoxins production in *P. aurantiogriseum* remain poorly understood. No studies have yet identified the gene clusters involved in the mycotoxin biosynthesis pathway of *P. aurantiogriseum,* and only one study has been conducted on Paclitaxel [63].

### 3.4. Regulation of Mycotoxin Biosynthesis

The availability of environment and nutrients to a fungal organism significantly impacts the production of its mycotoxins. The optimal conditions for metabolite production are not necessarily the same as those for optimal growth. The nature and quantity of mycotoxin produced by a fungus were closely impacted by the composition of the culture medium, as well as the availability of nutrients and additional factors such as carbon [64], nitrogen [65], phosphate [66], and other physicochemical parameters [60, 67]. In response to these factors, signals were generated and transmitted by proteins such as CreA in response to carbon [68], AreA in response to nitrogen [69], and PacC in response to changes in pH [70]. Numerous studies illustrate how oxidative stress modulates secondary metabolism, highlighting the importance of understanding the regulatory mechanisms involved in mycotoxin biosynthesis in filamentous fungi. The msnA protein regulates the signals generated in response to these stimuli [71]. For example, the production of terrestric acid, aurantiamine, and penicillic acid in *P. aurantiogriseum* [72, 73] is stimulated by oxidative stress. Figure 2 illustrates the potential connections between environmental signals and regulatory elements involved in different mycotoxin regulatory mechanisms.

## 4. Cancer Therapy by *P. aurantiogriseum* Mycotoxins

Mycotoxins represent a worldwide threat to human health and safety because of their toxicity and occurrence in food for humans and animals [74]. In addition, they are utilized in agriculture and medicine for their therapeutic application. Once biostudies establish their therapeutic effects, these isolated mycotoxins are called “bioactive molecules.” After structural modifications and biological testing, bioactive metabolites are models for developing new pharmaceutical drugs. Several studies have demonstrated the efficacy of mycotoxins from *P. aurantiogriseum* as antidiabetic, antibacterial, antioxidant, and insecticide agents [50]. In addition, numerous mycotoxins produced by various molds have shown antitumor properties in different cancer cell lines, both *in vitro* and *in vivo*, with some promoted as potential cancer treatments [75]. The interest in reviewing *P. aurantiogriseum* stems from its production of these mycotoxins.

The impact of mycotoxins extends their direct application by including their derivatives, such as semisynthetic analogs. Several mycotoxin derivatives have therapeutic applications and present significant positions in modern medicine. Numerous studies have explored mycotoxins and their derivatives for their anticancer effects, highlighting the pharmacological significance of this scaffold [76]. Studies have demonstrated the effectiveness of specific mycotoxin derivatives in treating cancer [77], and some have already been introduced to the market [31, 78]. In the following, we present key mycotoxins from *P. aurantiogriseum,* with certain analogs or derivatives that exhibit antioxidant and antitumor potential.

### 4.1. Anacin

**Figure 3:**
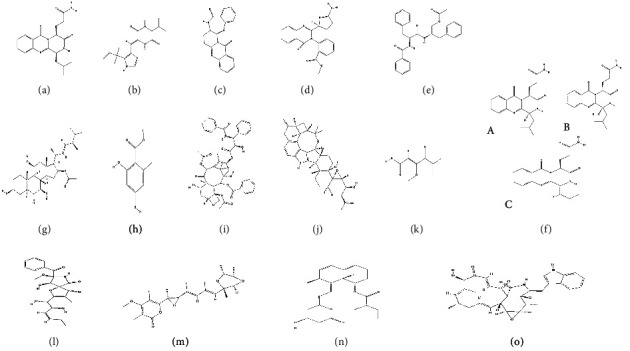
Chemical structure of *P. aurantiogriseum* mycotoxins (from PubChem database). (a) Anacin. (b) Aurantiamine. (c) Auranthine. (d) Auranomide B. (e) Aurantiamide acetate. (f) Aurantiomides A-C. (g) Anicequol. (h) Orsellinic acid. (i) Paclitaxel. (j) Penitrem B. (k) Penicillic acid. (l) Pseurotin A. (m) Verrucosidin. (n) Compactin. (o) Chaetoglobosin A.

Anacin, a quinazoline alkaloid first isolated from *P. aurantiogriseum* SP0-19 [79], has a revised structure, as reported by Larsen et al. [33] (Figure 3(a)). Its name derives from the involvement of anthranilic acid and leucine in its biosynthesis [80], along with L-glutarimide [81]. Patents have been field for the use of Anacin as a COX-2 inhibitor for the treatment, prevention, or inhibition of metastasis in patients with advanced breast cancer [82].

### 4.2. Aurantiamine

Aurantiamine (Figure 3(b)) is a cyclic dipeptide alkaloid known as a diketopiperazine containing a diketopiperazine ring composed of valine and histidine. This alkaloid was isolated from *P. aurantiogriseum* var. aurantiogriseum [33, 44]. Aurantiamine is a derivative of phenylahistin and belongs to a new class of colchicine-like microtubule-binding agents, exhibiting cytotoxic activity against a wide variety of tumor cell lines [83]. This process is called cell-cycle inhibition, explicitly targeting the G2/M phase [9].

### 4.3. Auranthine

Auranthine (Figure 3(c)), isolated in 1986 from *Penicillium aurantiogriseum*, is a fungal benzodiazepine. By successfully synthesizing (±)-auranthine, we confirmed the refined structure of natural (−)-auranthine. Studies have demonstrated that natural (−)-auranthine is a fused quinazolino benzodiazepine dione-1 that includes an acyclic aliphatic nitrile group, disproving the previously proposed structure [84].

### 4.4. Auranomide B

The alkaloid auranomide B (Figure 3(d)) was isolated from *Penicillium aurantiogriseum* MF361 by Xin et al. 2007 [79]. The chemical structure of this compound consists of quinazoline-4-one derivatives, a class of natural scaffolds that have been recognized as drug-like templates in medicinal chemistry and are favored for their structural properties [35]. Auranomide B exhibited moderate cytotoxic activity against the K-562, ACHN, HePG2, and A-549 cell lines, showing the highest activity against HePG2 cells [85].

### 4.5. Aurantiamides

Aurantiamides (Figure 3(e)) were isolated from *P. aurantiogriseum* [37]. Several aurantiamide dipeptide derivatives have recently demonstrated cytotoxic effects against cancer cells [83]. For example, peptichemio (PTC) [86] and bortezomib (Velcade) [87] have been developed and marketed for cancer treatment.

### 4.6. Aurantiomides A, B, and C

Three cytotoxic quinazoline alkaloids, aurantiamides A, B, and C, were first discovered in *P. aurantiogriseum* MF361 by Xin et al. [79]. Structurally, they are closely related to Anacin. Aurantiomide A, B, and C (Figure 3(f)) showed cytotoxicity effects against various cell lines [79]. In addition, there are patents related to aurantiomide dipeptide derivatives utilization to treat or prevent angiogenesis-related diseases, including patent US9872882B2, invented by Yeh et al. [31].

### 4.7. Anicequol

Anicequol (Figure 3(g)) was identified from *P. aurantiogriseum* Dierckx TP-F0213 [34]. Its structure is determined to be (3b, 5a, 7b, 11b, 16b, 24S)-16- acetoxy-3,7,11-trihydroxy-ergost-22-en-6-one, classifying it a sterol (oxysterol) [88]. Anicequol inhibits anchorage-independent growth of DLD-1 colon tumor cells by inducing anoikis (apoptosis). This process is associated with the p38 MAPK pathway, which acts as a suppressor of cell survival and tumorigenesis, and the ROCK pathway, which includes serine/threonine kinases that indirectly phosphorylate the light chain of myosin 2 (MLC2) [88].

### 4.8. Orsellinic Acid

Orsellinic acid (Figure 3(h)) was identified in *P. aurantiogriseum* [89] and serves as a precursor to penicillic acid [90]. The gene expression of the *ors* gene cluster (*orsa-e*) produces orsellinic acid [89]. Derivatives of orsellinic acids, such as azaphilone, armillyl, and 6-deoxyhexose, exhibit diverse biological activities, including antibacterial, antiviral, and antitumor action [91, 92]. In addition, they inhibit interleukin (IL) 1 and caspase-1 [93].

### 4.9. Paclitaxel

Taxol, also known as paclitaxel (10-deacetylbaccatin III), is a natural anticancer drug isolated from *P. aurantiogriseum* IHEM 14753 by El Jaziri and Diallo [46]. Paclitaxel (Figure 3(i)) is currently used to treat lung, breast, and ovarian cancer, as well as Kaposi's sarcoma. It is sometimes combined with other drugs such as cisplatin or carboplatin. In addition, it is used off-label for cancers of the cervix, gastroesophageal tract, endometrium, prostate, and area of the head and neck, along with lymphoma, leukemia, and sarcoma. Paclitaxel is well-known for causing the mitotic arrest, leading to cell death in a portion of the arrested cell population [78].

### 4.10. Penitrem

Penitrem B (Figure 3(j)) is a diterpene mycotoxin isolated from *P. aurantiogriseum* by Wilson et al. [93]. This mycotoxin has been reported to exhibit *in vitro* inhibition of proliferation, migration, and invasion in human breast cancer cells [94]. Penitrem B demonstrated *in vitro* growth inhibition of the human leukemia cell line in a screening test [94, 95].

### 4.11. Penicillic Acid

Penicillic acid (Figure 3(k)) is a polyketide mycotoxin isolated from *P. aurantiogriseum* [43, 44, 96]. It exhibits vigorous antitumor activity by inhibiting the cell division of mammalian cells [97, 98]. In addition, penicillic acid demonstrates pharmacological effects, including blood vessel dilation and antidiuretic properties [99].

### 4.12. Pseurotin

Pseurotin A (Figure 3(l)) is produced via a mixed biosynthetic pathway that couples phenylalanine with a hexaketide and forms a spiro ring structure. It was first isolated from *P. aurantiogriseum* by Frisvad and Lund [25]. Pseurotin analogs were shown to have angiogenesis-inhibitory activity [34], and in 2012, four pseurotin analogs were demonstrated to be effective against the human breast cancer cell line MCF-7 [9]. Azaspirene, a related compound in the pseurotin family, represents a novel anticancer agent that inhibits angiogenesis by blocking the blood supply signals sent by tumor cells rather than targeting cancer cells directly, as in traditional chemotherapy [98].

### 4.13. Verrucosidin

Verrucosidin (Figure 3(m)) was isolated from *P. aurantiogriseum* MF361 [44] and has been associated with outbreaks of neurological diseases due to its inhibition of oxidative phosphorylation in mitochondria [100]. It acts as a downregulator of UPR-induced genes, such as *grp78*, leading to selective cell death under strict hypoglycemic conditions. In addition, two new analogs of verrucosidin (verrucosidinol [101] and verrucosidinol acetate [102]) were isolated from this marine-derived fungus [22], along with norverrucosidin [4] and verrucosidin [5].

### 4.14. Compactin

Compactin (Figure 3(n)), also known as ML-236B, is a nonaketide isolated from *P. aurantiogriseum* [38]. It acts as a potent inhibitor of 3-hydroxy-3-methylglutaryl coenzyme A (HMG-CoA) reductase [103] and is commonly used in clinical settings for cholesterol-lowering purposes [104]. At higher concentrations, Compactin was shown to reduce pigmentation and inhibit the proliferation and invasion of melanoma cells [105]. The compound is protected under U.S. Patent no. 3983140, issued on September 1977 [103].

### 4.15. Chaetoglobosin A

Chaetoglobosin A (Figure 3(o)), a member of the cytochalasin alkaloids group, contains a 10-(indol-3-yl) group, a macrocyclic ring, and a perhydroisoindolone moiety [106]. It was isolated from *P. aurantiogriseum* [107]. Studies showed that chaetoglobosins exhibit significant biological activities, including antitumor, antifungal, phytotoxic, fibrinolytic, antibacterial, nematicidal, anti-inflammatory, and anti-HIV properties [106, 108].

## 5. Underlying the Mechanisms of Action of *P. aurantiogriseum* Mycotoxins

**Figure 4:**
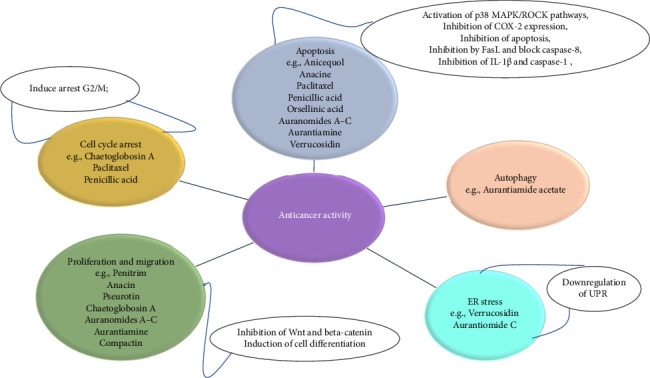
Anticancer pathways of some *P. aurantiogriseum* mycotoxins.

Chemotherapy has been proposed to control recognized malignancies. Multiple substances that are effective for treating various conditions have acted typically by killing rapidly dividing cells and, since they are not selective, also kill healthy cells. This has been the main drawback of chemotherapy since it causes severe secondary effects [23]. Therefore, growing interest was observed for targeted therapies using anticancer agents specifically attacking abnormal cells and drawing inspiration from fungal metabolites that have shown promise in preventing and treating cancer. These compounds have become more interesting because of their anti-inflammatory and antioxidant activities, which are supported by their biological effects. The contribution of these natural chemicals extends beyond their direct application in unmodified structures to include their derivatives, such as semisynthetic analogs of lead structures and synthetic structural mimics. Several mycotoxins produced by *P. aurantiogriseum* have recently demonstrated anticancer solid effects. These effects were mediated by several mechanisms, including inhibiting crucial enzymes, stimulating death pathways, or promoting growth arrest (Figure 4).

Several studies demonstrated the effectiveness of mycotoxins derived from *P. aurantiogriseum* in fighting cancer cells through various mechanisms and activities (Table 3). Anacin, a mycotoxin produced by *P. aurantiogriseum,* is marketed not only as a pain reliever but also shows potential in cancer therapy by inhibiting COX-2 synthesis and modulating cellular proliferation and apoptosis [129]. Anicequol, another mycotoxin from *P. aurantiogriseum,* exhibits cytotoxicity across multiple cancer cell lines by inducing apoptosis through the p38 MAPK and ROCK pathways, effectively suppressing cell survival and tumorigenesis [130]. Auranomide B acts as a kinesin spindle protein (KSP) inhibitor, showing potent cytotoxicity against HepG2 cells [131]. Aurantiamide acetate inhibits lysosomal protease degradation, causing mitochondrial fragmentation and decreased cell viability in glioma cells; it is related to marketed drugs such as PTC and bortezomib [132]. Aurantiamine, similar to colchicine, induces G2/M cell-cycle arrest, halting proliferation in various tumor cell lines and exhibiting broad-spectrum cytotoxicity [130]. Aurantiomide C shows potent cytotoxic and antioxidant activities against cancer cells such as HL-60 and HepG2 [131]. Chaetoglobosin A inhibits actin polymerization and cell migration and sensitizes cells to kinase inhibitors, inducing G2/M arrest, which is effective against several cancer cell lines [133]. Compactin, or mevastatin, inhibits melanoma cell growth and proliferation at high doses [134]. Orsellinic acid, by inhibiting IL 1 and caspase, reduces apoptosis in cancer cells such as NCI-H460 [135]. Paclitaxel, widely used in cancer treatment, stabilizes tubulin polymerization, induces G2/M arrest, and promotes apoptosis by inhibiting Bcl-2, which is essential for treating ovarian, breast, and lung cancers [131]. Patulin induces apoptosis in colon cancer cells by generating reactive oxygen species (ROS) and activating the mitochondrial pathway [136]. Penicillic acid inhibits caspase-8 and FasL, showing cytotoxic effects on Raji and HeLa cells and disrupting cell division [137]. Penitrem B, by inhibiting BK channels in the Wnt/*β*-catenin pathway, shows antiproliferative and antimigratory activities, especially in breast cancer cells [138]. Pseurotin inhibits cell proliferation [139], which is linked to a decrease in the expression of cyclins and mitochondrial respiration through the specific inhibition of signal transducer and activator of transcription 3 (STAT3) phosphorylation. It significantly inhibited the production of nitric oxide and tumor necrosis factor, as well as the expression of inducible NO synthase (iNOS) and IL-6 [140]. Verrucosidin downregulates UPR-induced genes, leading to selective cell death under hypoglycemic conditions. Verrucosidinol acetate, despite its low cytotoxicity, is explored for its novel chemical framework in cancer therapy [131].

## 6. Advantages and Limitations

Recent advancements in cancer therapies have emerged from the development of various natural compounds. Numerous studies have suggested exploring the marine environment as a source of pharmacologically active secondary metabolites [12, 28, 141, 142]. Marine fungi are crucial for developing new medications and providing fresh scaffolding that can be further altered to produce the desired effect. Beginning with the well-known example of cephalosporins, marine fungi have produced distinctive chemical skeletons that may be exploited to create medicines of clinical value [143–145]. Exploration of marine environments has become increasingly important for drug discovery, with several FDA-approved medications in recent decades originating from marine sources. Most marine-based medications are sourced from the sponge, tunicate, mollusk, and bryozoan invertebrates. Two-thirds of these medications fall under the category of nonribosomal peptides. Some of these are already available on the market as antibiotics and anticancer medications (Polymyxin B, pristinamycin, gramicidin, vancomycin, bleomycin, and actinomycin D), while others are still in clinical testing (manoalide and discodermolide) [146]. As previously mentioned, *P. aurantiogriseum* is a marine facultative fungus that thrives in marine and terrestrial environments, making the biosynthesis of its metabolic products dependent on environmental, physical, and biological factors. Consequently, even small changes to these factors may produce an entirely new set of metabolic products [85, 147]. In addition, the use of mycotoxins has several advantages for the treatment and prevention of cancer. Mycotoxin production is often considered safe and straightforward, with relatively small quantities recommended for treatment. Storage and transport are not complicated [148]. *P. aurantiogriseum* contains the majority of mycotoxins with demonstrated anticancer effects, making it the most promising for cancer treatment. Some have already been marketed as pharmaceutical chemical agents. However, our understanding of the precise benefits and drawbacks of employing mycotoxins in cancer treatment is currently limited.

On the other hand, recent advances show that mycotoxins can be combined with chemotherapy agents to result in synergistic effects, increasing cancer cell death and decreasing drug resistance [149, 150]. Through nanotechnology, targeted delivery systems have been achieved, wherein mycotoxins are entrapped within nanoparticles that could enhance bioavailability and diminish toxicity to normal cells, thus enabling them to reach the tumor site effectively for richer therapeutic potential [151, 152]. However, they still face some challenges, including their potential toxicities, stability, and other untoward effect possibilities. In future studies, more optimization should be performed with drug delivery systems, understanding the exact mechanism of action and carrying out clinical trials required to determine the safety and efficacy of mycotoxins in cancer therapy.

## 7. Other Applications for *P. aurantiogriseum* Mycotoxins

### 7.1. In the Food Industry

*P. aurantiogriseum* plays an essential role in the maturation of certain fermented products [153]. It offers a pleasant appearance and participates in maturation through its extracellular proteases and lipases [33, 154, 155]. *P. aurantiogriseum* showed not only intense proteolytic and lipolytic activities but also played a crucial role against lipid oxidation in fermented products by producing enzymes such as deaminases that could help break down amino acids and form compounds that could contribute to the characteristic flavor and aroma of the final product [156].

### 7.2. In the Agricultural Industry

Several studies have shown the antibacterial and antifungal effects of *P. aurantiogriseum* [11, 114]. *P. aurantiogriseum* was tested against four bacterial pathogenic strains (*Staphylococcus aureus*, *Bacillus cereus*, *B. subtilis*, and *Salmonella* sp.). Similarly, Mustapha et al. reported that *B. subtilis* was most sensitive to *P. aurantiogriseum* culture filtrate and that *Staphylococcus typhimurium* was slightly sensitive [11]. In the same order, *P. aurantiogriseum* was tested against four fungal strains: *Aspergillus ochraceus*, *A. flavus, Fusarium solani,* and *Alternaria alternata* [114]. In addition, *P. aurantiogriseum* mycotoxins have proven to have antibacterial and antifungal properties. Auranomide B exhibited methicillin-resistant *Staphylococcus aureus* (MRSA, Clinical isolates), *Candida albicans,* and synergistic antifungal activity with ketoconazole [35]. Aurantiamides displayed antibacterial, anti-inflammatory, antioxidant, and anti-HIV effects [157]. Penicillic acid showed antibacterial properties (Oxford, 1942) [97, 158]. Pseurotin A displayed antifungal activities [32]. Also, *Staphylococcus aureus*, *Xanthomonas campestris* var. *vesicatoria* and *Ralstonia solanacearum* were most sensitive to orsellinic acid [159]. Compactin has been reported to exhibit antifungal properties [160]. Chaetoglobosin A has demonstrated potent inhibitory effects on various fungal species, including *Mucor miehei, Setosphaeria turcica, Botrytis cinerea*, *Sclerotinia sclerotiorum*, *Rhizopus stolonifer*, and *Coniothyrium diplodiella* [161]. In addition, orsellinic acid exhibits antibacterial activity against *Staphylococcus* species [162].

### 7.3. In the Pharmaceutical Industry

Several studies have reported the effect of mycotoxins in the pharmaceutical field. Aurantiamides, aurantiomides, and their analogs exhibited anti-inflammatory, antioxidant, and anti-HIV effects [163, 164]. In addition, penicillic acid showed antiviral and antitumor activity [97, 158]. Compactin inhibited HMG-CoA reductase activity in tissue culture cells by inhibiting cholesterol synthesis and reducing plasma cholesterol levels in dogs, primates, and humans [165]. Orsellinic acid demonstrated antioxidant efficacy in a *β*-carotene–linoleate model system [162].

## 8. Conclusion

*P. aurantiogriseum* mycotoxins play a significant role in illness investigations that impact people, animals, and crops. The discovery of these mycotoxins, which have antitumor effects, offers new therapeutic targets for cancer. *P. aurantiogriseum* possesses a staggering number of mycotoxins, with at least 15 identified and documented as having anticancer potential, making it a promising candidate for future anticancer treatments. Combining mycotoxins and chemotherapy can have either synergistic or antagonistic effects on cell viability, highlighting the need to investigate their interactions in toxicological assessments. Nanoparticle-based mycotoxins represent a next-generation approach to anticancer therapy, enhancing bioavailability while reducing toxicity to normal cells. However, limited research in this area necessitates further investigation. Several technological approaches hold promise for biochemical and molecular research to identify and validate these compounds. A thorough research is required to fully comprehend the functional significance of these anticancer mycotoxins and develop more effective therapeutic approaches. This includes optimizing delivery systems, determining precise mechanisms of action, and performing clinical trials to assess the safety and efficacy of cancer therapy.

## Figures and Tables

**Table 1:** *Penicillium aurantiogriseum* mycotoxins adapted by Frisvad et al. [32].

Mycotoxins	Chemical formula	Bioactivity	References
Anacin	C_18_H_22_N_4_O_3_	Nonopioid analgesic and antipyretic drug	[33]

Anicequol	C_30_H_48_O_6_	Inhibitor of tumor cell growth	[34]

Auranomide B	C_20_H_18_N_4_O_3_	Cytotoxic	[35]

Auranthine	C_19_H_14_N_4_O_2_	Cytotoxic	[14, 36]

Aurantiamides acetate	C_27_H_28_N_2_O_4_	Cytotoxic	[37]

Aurantiamine	C_16_H_22_N_4_O_2_	Cell-cycle inhibitor	[38]

Aurantiomides A-C	C_19_H_24_N'O_4_ C_18_H_22_N_4_O_4_ C_18_H_20_N_4_O_3_	Cytotoxic	[35]

Chaetoglobosin A	C_32_H_36_N_2_O_5_	Antibacterial, antifungal, phytotoxic, anticancer, and cytotoxic	[39, 40]

Compactin	C_23_H_34_O_5_	Cholesterol-lowering	[41]

Orsellinic acid	C_8_H_8_O_4_	Antioxidant, antibacterial, block PAF-mediated neuronal apoptosis	

Patulin	C_7_H_6_O_4_	Immunosuppressant: lymphopenia; neurotoxic: antiacetylcholinesterase action	[42]

Penicillic acid	C_8_H_10_O_4_	Antibiotic, antiviral, cytotoxic, and carcinogenic	[43, 44]

Penitrem B	C_37_H_45_NO_5_	Antibiotic and cytotoxic	[45]

Pseurotin A	C_22_H_23_N_5_O_2_	Angiogenesis inhibitors, inhibitors of immunoglobulin E, and antifungal	[25]

Roquefortine C and D	C_22_H_23_N_5_O_2_ C_22_H_25_N_5_O_2_	Antibiotic and neurotoxin	[21]

Taxol	C_47_H_51_NO_14_	Anticancer drug	[46]

Terrestric acid	C_11_H_14_O_4_	Cardiotoxin	[43]

Verrucisidinol and verrucosidinol acetate	C_24_H_34_O_7_ C_26_H_36_O_8_	Tremorgenic and cytotoxic	[22, 47]

Verrucosidin	C_24_H_32_O_6_	Tremorgenic	[44, 45, 48]

**Table 2:** Molecular categories of mycotoxins from *P. aurantiogriseum* (adapted and modified from Bu'lock et al. [59]).

Molecular categories	Mycotoxins	References
Polyketides	Terrestric acid	[54, 59]
Tetra-	Penicillic acid, orsellinic acid, and patulin	[11, 59]
Hexa-	Pseurotin	[54, 59]
Nona-	Verrucosidin and Compactin	[11, 59]
Deca-	Chaetoglobosin A	[54, 59]
Terpenes		
Di-	Penitrem and paclitaxel	[55, 59]
Tri-	Anicequol	[56, 59]
Meroterpene	Roquefortine C	[54, 57, 59]
Peptides	Anacin, auranthine, aurantiamine, and aurantiamides	
Alkaloids	Auranomide B	
Hybrids	Chaetoglobosin A and terrestric acid	

**Table 3:** Profiles of *P. aurantiogriseum* mycotoxins in cancer therapy.

Mycotoxins measured	Drug	Cell lines	Signaling pathways	IC 50% (μM)	Findings	References
Anacin	Anacin and many other brand names	Clinical use: treat cancer pain	Inhibition of COX-2 synthesis	—	Inhibit tumor development and growth by modulating cellular proliferation and apoptosis.	[32, 39, 109, 110]

Anicequol	—	DLD-1, K-562, MCF-7, HeLa, DU145, U-937, H-1975, SGC-7901, A-549, MOLT-4, and HL-60	Induces apoptosis by activation of the p38 MAPK and ROCK pathways	1.2, 11.6, 17.0, 12.9, 7.37, ≥50, 13.1, 35.0 ≥ 50, 6.53, and 1.75	Suppressor of cell survival and tumorigenesis inhibits the anchorage-independent growth of colon tumor cells DLD-1	[34, 88, 111]

Auranomide B	—	HepG2	KSP inhibitors	0.097	Cytotoxic	[35]

Aurantiamide acetate	Peptichemio (PTC) and Bortezomib (Velcade®)	Clinical use	Inhibition of lysosomal protease degradation induces moderate mitochondrial fragmentation	35	Decreased cell viability, inhibited intracellular autophagic flux, and lost mitochondrial membrane potential.	[39, 112, 113]

Aurantiamine	Like substance colchicine	A-431, A-549, HeLa, K-562 and P-388, MCF-7, TE-671, and WiDr	Stops cell proliferation and induces G2/M cell-cycle arrest	0.18–3.7	Cytotoxic	[9, 12, 38, 114]

Aurantiomides A-C	—	HL-60, P-388, BEL-7402, P-388, HePG2, MCF-7, HCT-116, HeP2, HeLa	—	52 μg, 54, 25, 10–50, 23.94, 20.48, 9.51, 37.63, and 18.47	Potent cytotoxic and antioxidant activity	[79, 115]

Chaetoglobosin A	—	CLL, SGC-7901, A-549, HTC-116, and HeLa	Induces G2/M cell-cycle arrest inhibition of CLL migration activation and sensitizes cells for treatment with kinase inhibitors	2.8, 7.48 6.56, 3.15, 3.2	A unique inhibitory activity against actin polymerization in mammalian cells	[9, 116, 117]

Compactin	Mevastatin	LMA		IC 100%: 2 and 6	High doses inhibit the growth and proliferation of melanoma cells	[39]

Orsellinic acid	—	NCI-H460, SF-268	Inhibition of interleukin 1 and caspase (key actor in apoptosis)	6,5, 8,8	Attenuates G-protein coupled receptor (PAFR) independent neuronal apoptosis	[118–120]

Paclitaxel	Paclitaxel, Pacliwell, Taxol, Paclitero, Geltax…	Commonly used to treat ovarian, mammary, pulmonary, lung tumors, and Kaposi's sarcoma	Induces cell G2/M cycle arrest apoptosis through promoting and stabilizing tubulin polymerization	—	Act against mitochondria and inhibit apoptosis inhibitor protein b-cell leukemia 2 (Bcl-2)	[75, 121, 122]

Penicillic acid	—	Raji cells, mammalian cells, and leukemia SN-36	Inhibits apoptosis by FasL, blocks self-processing of caspase-8, and inhibits cell division	100–200, 5	Loose cell viability in Raji cells of lymphoma Burkitt. Slow action on HeLa cells Accumulation of metaphase cells, elongation of the whole cell cycle	[97, 114, 123, 124]

Penitrim	—	MCF-7, MDA-MB-231, A-549, and HL-60	Inhibits the BK channels of the Wnt/b-catenin pathway (a key factor in the migratory and invasive potential of breast cancer cells)	11.9, 9.8, 4.6, and 2.6	Antiproliferative and antimigratory	[85, 125, 126]

Pseurotin A	—	K-562, MCF-7, and HCT-116	Inhibits the IgE production and inhibits cell proliferation; inhibition of monoamine oxidase exhibiting potential antiproliferative activity	3.6, 15.6, 72.0	Apomorphine and antiangiogenic activity	[114, 127, 128]

Verrucosidin Verrucosidinol acetate	— —	HT-29, HeLa, MDA-MB-231, MCF-7, MGC-803, and A-549	Downregulation of UPR-induced genes resulting in selective cell death under strict hypoglycemic conditions	50 nM, 3.91, 1.91, 3.80, 1.84, and 2.50 >10.0	Prevents UPR-induced expression of proteins such as GRP78, triggered by glucose deprivation or 2-deoxyglucose Cytotoxic	[16] [47]

*Note:* MCF-7, breast cancer cell line; HepG2, liver hepatocellular carcinoma cell line; HL-60, leukemia cell line; NCI-H460, non-small cell lung cancer cell line; DLD-1; colorectal adenocarcinoma cell line; K-562, immortalized myelogenous leukemia cell line; Hela, cervical cancer cell line; DU145, prostate cancer cell line; U-937, myeloid leukemia cell line; H-1975, lung cancer cell line; SGC-7901, gastric cancer cell line; A-549, lung cancer cell line; MOLT-4, T lymphoblast cell line; A-43, epidermoid carcinoma cell line; P-388, mouse lymphoid neoplasm cell line; TE-671, rhabdomyosarcoma cell line; WiDr, colon adenocarcinoma cell line; BEL-7402, hepatocellular carcinoma cell line; HCT-116, colon cancer cell line; HeP2, (hepatitis B 2) cervical carcinoma cell line; SGC-7901, gastric cancer cell line; MDA-MB-231, breast cancer cell line; MGC-803, gastric carcinoma cell line; HT-29, colorectal adenocarcinoma cell line; SF-268, glioblastoma cell line; Caspase-8, FLICE (FADD-like interleukin-1*β*-converting enzyme); BK channels, large conductance Ca2+-activated K+ channels; Wnt/*β*-catenin pathway, signaling system including embryonic development, cell proliferation, differentiation, and migration.

Abbreviations: CLL, chronic lymphocytic leukemia; COX-2, cyclooxygenase-2; FasL, fast ligand (signaling molecule); GRP78, glucose-regulated protein 78; KSP, kinesin spindle protein; LMA, acute myeloid leukemia; p38MAPK, p38 mitogen-activated protein kinase; PAF, platelet-activating factor; ROCK, rho-associated protein kinase; UPR, unfolded protein response.

## Appendix A

Not applicable.
